# Synergistic Dual Ag/Cu Ion Implantation to Enhance Antimicrobial Defense on Boston Keratoprosthesis

**DOI:** 10.34133/bmr.0147

**Published:** 2025-02-19

**Authors:** Silvia G. Gómez, Gerard Boix-Lemonche, Jordi Orrit-Prat, Raül Bonet, Jaume Caro, Joan Muñoz, Maria-Pau Ginebra, Barbara Skerlavaj, Rafael I. Barraquer, José M. Manero

**Affiliations:** ^1^ Biomechanics, Biomaterials and Tissue Engineering, Department of Materials Science and Engineering, Universitat Politècnica de Catalunya · BarcelonaTech (UPC), Barcelona 08019, Spain.; ^2^ Barcelona Research Centre in Multiscale Science and Engineering, Universitat Politècnica de Catalunya · BarcelonaTech (UPC), Barcelona 08019, Spain.; ^3^ Center for Eye Research and Innovative Diagnostics, Department of Ophthalmology, Institute of Clinical Medicine, Faculty of Medicine, University of Oslo, Oslo 0450 Norway.; ^4^ Eurecat, Centre Tecnològic de Catalunya, 08243 Manresa, Spain.; ^5^ Microdent Implant System S.L., 08187 Santa Eulàlia de Ronçana, Spain.; ^6^ Centro de Investigación Biomédica en Red—Bioingeniería, Biomedicina y Nanomedicina (CIBER-BBN), Madrid, Spain.; ^7^ Institute for Bioengineering of Catalonia (IBEC), Barcelona Institute of Science and Technology, 08028 Barcelona, Spain.; ^8^ Department of Medicine, University of Udine, 33100 Udine, Italy; ^9^ Centro de Oftalmología Barraquer, Barcelona, Spain.; ^10^ Institut Universitari Barraquer, Universitat Autònoma de Barcelona, Barcelona, Spain.; ^11^ Universitat Internacional de Catalunya, Barcelona, Spain.

## Abstract

The Boston keratoprosthesis (BKPro) is a critical device for vision restoration in complex cases of corneal blindness, although its long-term retention is challenged by infection risks. This study aims to enhance the antimicrobial properties of the titanium (Ti) backplate of the BKPro by ion implanting silver and copper ions to achieve effective infection control while maintaining cytocompatibility. Research on antimicrobial modifications for BKPro is limited, and while metallic ions like Ag and Cu show promise for biomaterial improvement, their effects on human corneal keratocytes (HCKs) require further study. Ag and Cu were implanted onto rough Ti surfaces, as mono- and coimplantations. Cytotoxicity was assessed in HCKs, and antimicrobial efficacy was tested against *Pseudomonas aeruginosa* and *Candida albicans*. After 21 d, monoimplanted Ag samples released 300.4 ppb of Ag^+^, coimplanted samples released 427.5 ppb of Ag^+^ and 272.3 ppb of Cu ions, and monoimplanted Cu samples released 567.0 ppb of Cu ions. All ion-implanted surfaces supported HCK proliferation, exhibited no cytotoxicity, and showed strong antimicrobial activity. Ag-implanted surfaces provided antibacterial effects through membrane disruption and reactive oxygen species generation, while Cu-implanted surfaces exhibited antifungal effects via impaired enzymatic functions and reactive oxygen species. Coimplanted AgCu surfaces demonstrated synergistic antimicrobial effects, resulting from the synergy between the bactericidal actions of Ag and the oxidative stress contributions of Cu. Additionally, ion-implanted surfaces enhanced HCK adhesion under co-culture conditions. In conclusion, ion implantation effectively imparts antimicrobial properties to the Ti backplate of BKPro, reducing infection risks while preserving compatibility with corneal cells.

## Introduction

According to the World Health Organization, approximately 36 million people worldwide are blind, with around 4.2 million suffering from corneal blindness [1]. Corneal disease ranks as the fifth leading cause of blindness, following conditions such as cataracts, glaucoma, and age-related macular degeneration [2]. Corneal blindness occurs due to the compromised integrity or transparency of corneal tissue, leading to vision loss despite the otherwise normal functioning of ocular structures [3].

Penetrating keratoplasty, or corneal grafting, is the gold-standard treatment for corneal blindness. However, not all patients are suitable candidates for this procedure, including those with a history of corneal graft rejections, advanced autoimmune disease, or acute inflammation [4]. Treatment for this group of high-risk patients involves the use of artificial corneas, also known as keratoprostheses (KPros). Over the past few decades, there has been a notable increase in the use of KPros to restore vision, with the Boston keratoprosthesis (BKPro) emerging as the preferred choice, having been implanted in over 15,000 individuals worldwide [5,6]. While short-term outcomes following BKPro implantation are promising, concerns are growing over infections, which can significantly impair visual acuity and long-term device success.

Bacterial and fungal infections pose substantial risks to ocular health, resulting in corneal opacity, inflammation, graft failure, and vision loss. In BKPro recipients, infection rates range from 3.4% to 21.4% [7], with *Pseudomonas aeruginosa* and *Candida albicans* identified as common pathogens [8]. Current treatment methods rely heavily on pharmacological interventions, including complex regimens of topical and oral antibiotics. Despite rigorous adherence to hygiene and medication protocols, these treatments often fail to completely eradicate established infections, and there is no standardized treatment, with various drug combinations being used [5].

To prevent infections in implants, researchers have explored various surface modification strategies, including antiadhesive techniques, contact-killing mechanisms, controlled drug release, and the development of materials with intrinsic antimicrobial properties [9]. However, these strategies have notable drawbacks. Antiadhesive techniques may interfere with the proper biointegration of implants, while contact-killing mechanisms are ineffective against bacterial infections that occur before direct contact. Controlled drug release systems present often an initial “burst release”, which fails to provide sustained antimicrobial effects over time, and materials with intrinsic antimicrobial properties are more expensive to produce [10].

The rise of microbial resistance underscores the need for multifaceted modifications capable of combating infections through diverse strategies simultaneously. Metal ions, such as essential ones like copper (Cu) and iron (Fe), and nonessential ions like silver (Ag) and mercury (Hg), offer promising antimicrobial potential [11]. Essential metal ions play critical roles in cellular processes, including the formation of cell membranes and DNA. However, excessive concentrations can be toxic [12]. Nonessential metal ions exhibit toxicity even at low levels. The selective action of metal ions is attributed to differences in metalloproteins and metal transport systems, allowing differentiation between mammalian cells and microorganisms [13].

Research dedicated to infection prevention in BKPro remains limited, with current studies focusing on functionalizing implant surfaces with biomolecules [14]. Despite the promising results obtained, the use of biomolecular coatings faces challenges such as stability, proteolytic degradation, and high production costs [15]. Consequently, there is a critical need to develop effective strategies that confer intrinsic microbial defense to the BKPro.

Silver and its ions are renowned for their antimicrobial properties against a broad spectrum of microorganisms, including bacteria, fungi, and viruses. Despite extensive research, the exact antimicrobial mechanism of silver remains incompletely understood, involving processes such as membrane disruption, interference with intracellular proteins and DNA, and generation of reactive oxygen species (ROS) [16]. In ophthalmology, Ag has been utilized in multiple applications, such as artificial eyes, intraocular lenses, topical antibacterial agents, and the diagnosis and treatment of ophthalmic diseases [17].

Copper has also emerged as an attractive antimicrobial agent due to its effectiveness against pathogens. Cu serves as a crucial cofactor in various biological processes depending on its oxidation state [18]. Like silver, the exact antimicrobial mechanism of Cu remains unclear, but it is believed to disrupt cell membranes, alter intracellular biochemical processes, and cause DNA damage [19]. The use of copper in combination with biomaterials has been highlighted as a promising strategy for the success of implants. In ophthalmology, Cu has been used as a nutritional supplement to prevent age-related macular degeneration [20].

The combination of Ag and Cu ions holds promise due to their potent antimicrobial properties and complementary mechanisms of action [11]. Synergistic effects between both ions could lead to the development of durable antimicrobial strategies with broad efficacy [21]. Additionally, combining these ions may enable dose reduction while ensuring enhanced biological compatibility [22].

Preventing infections in the early stages of biomedical implantation is crucial to inhibit microbial adhesion and subsequent biofilm formation, given the difficulty of eradicating established biofilms [23]. Developing antimicrobial strategies with sustained efficacy is equally important, not only to prevent initial microbial colonization but also to mitigate antimicrobial resistance and reduce repeated medical interventions [24]. Co-culture experiments, particularly those simulating preimplantation infection conditions, are essential for evaluating the effectiveness of biomaterials with antimicrobial properties [25]. These models provide a more accurate prediction of in vivo performance by comprehensively assessing the ability of antimicrobial treatments to prevent microbial adhesion and biofilm formation and their capacity to maintain antimicrobial efficacy over time [26]. Additionally, co-culture conditions yield valuable insights into the behavior of treated materials in balancing microbial control with biocompatibility. Despite the ongoing challenges related to implant-associated infections, the application of such approaches remains underexplored in the context of KPros, underscoring the need for further investigation into infection-resistant surfaces with sustained antimicrobial effects.

Ion implantation is a surface modification process based on accelerating ions that are impacted onto a surface, causing them to embed into that surface. The presence of these ions modifies the surface properties, improving material properties such as hardness and corrosion resistance [27]. Ion implantation continues to be the subject of research and refinement to optimize its application in biomedical implants.

Metal vapor vacuum arc (MEVVA) ion implantation is a technique used to embed high-energy metal ions into a target material. This process relies on a vacuum arc discharge to vaporize a metal, creating a plasma of metal ions that are accelerated toward a substrate [28]. This allows precise control of ion species, dose, and energy, making it ideal for enhancing surface properties such as antimicrobial resistance, hardness, and wear and corrosion resistance. The versatility and precision of MEVVA make it a valuable tool in materials science and biomedical engineering, especially for improving the functionality of medical implants and devices.

This study explored the feasibility of enhancing BKPro Ti backplates through MEVVA ion implantation. Given that the BKPro backplate has a rough topography, it was essential to evaluate both the effects of the ion implantation technique alone and its interaction with the rough surface. To achieve this, rough samples were prepared using sandblasting, referred to as “SB” throughout the article, to mimic the commercial BKPro. Rough samples were extensively characterized and used for all biological experiments. Physicochemical characterization assessed the changes on the treated surfaces, while biological evaluations confirmed that treated surfaces did not harm human corneal keratocytes (HCKs), which are vital for maintaining corneal stromal integrity. Additionally, the antimicrobial activity of the rough samples was tested against *P. aeruginosa* and *C. albicans*, 2 common pathogens involved in ocular infections.

This study has developed ion-implanted surfaces with antimicrobial properties and compatibility with HCKs. This research marks an advancement in preventing infections linked to KPros, potentially lowering healthcare burdens and enhancing patient outcomes.

## Materials and Methods

### Experimental design

#### Objective of the study

The main objective of this study is to use the MEVVA ion implantation technique to monoimplant Ag and Cu or coimplant Ag and Cu onto rough Ti surfaces. The goal is to obtain surfaces that are biocompatible with HCKs and nontoxic and simultaneously exhibit antimicrobial properties.

#### Study design

The study design includes 4 groups of samples:

• control SB: rough Ti surfaces without any treatment
• Ag samples: rough Ti surfaces monoimplanted with Ag ions
• Cu samples: rough Ti surfaces monoimplanted with Cu ions
• AgCu samples: rough Ti surfaces coimplanted with Ag and Cu ions

**Fig. 1.**
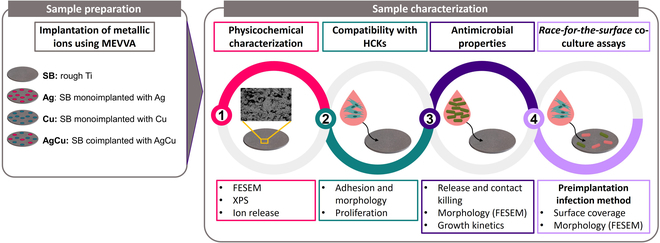
Scheme illustrating the workflow of this project. MEVVA, metal vapor vacuum arc; HCKs, human corneal keratocytes; FESEM, field emission scanning electron microscopy; XPS, x-ray photoelectron spectroscopy.

Each sample was subjected to the following experimental stages as depicted in the workflow of the project (Fig. 1):

1. MEVVA ion implantation: monoimplantation or coimplantation of Ag and Cu onto the selected samples
2. Physicochemical characterization: evaluation of the surface changes induced by the ion implantation treatment
3. Biocompatibility assays: analysis of cell adhesion, morphology, and proliferation to confirm compatibility with HCKs
4. Study of antimicrobial properties: assessment over short and long periods against *P. aeruginosa* and *C. albicans*
5. Co-culture assays: simulation of a more realistic scenario to evaluate the performance of the samples under preimplantation infection conditions
6. Data analysis and statistical evaluation: comprehensive analysis of the obtained results

### Sample preparation and cleaning

Commercially pure titanium grade 5 rods were machined to produce disks (10 mm in diameter, 2 mm in thickness). The obtained disks were ultrasonically cleaned using osmotized water and soap. The disks were sandblasted, using alumina (Al_2_O_3_) particles of 100 mesh, to obtain rough sandblasted surfaces (SB), similar to those found in commercial BKPro (Ra 1.9 ± 0.3 μm). The sandblasting conditions were as follows: distance of 5 cm, pressure of 2.5 bar, and duration of 90 s. Afterward, the disks were ultrasonically cleaned with osmotized water and soap. The ultrasonic cleaning process was carried out using distilled water, ethanol, and acetone, with each step lasting 5 min and repeated 3 times. Afterward, the samples were dried and stored.

### Ion implantation

Ag and Cu were implanted into the previously prepared disks using the MEVVA ion implantation technique. The ion implantation process was carried out by obtaining Ti substrates implanted with Ag and Cu ions individually (monoimplanted samples) and with both ions (coimplanted samples). For this purpose, 100Ag at. % (99.9% purity), 100Cu at. % (99.9% purity), or 60Ag40Cu at. % (99.9% purity) cathodes were evaporated using a pulsed cathodic arc source (MEVVA50, Plasma Technology Limited, China). In this process, the vacuum chamber was pumped down to a pressure below 5 × 10^−3^ Pa before conducting the ion implantation.

The samples were placed on a grounded substrate holder at room temperature. The acceleration ion implantation voltage was 20 kV, and the mean beam current was about 5 mA. The incident ion dose was estimated to be 4.3 × 10^17^ ions/cm^2^.

The sandblasted, nonimplanted control samples are called SB samples. For monoimplanted samples, those with silver are labeled as Ag samples, and those with copper are labeled as Cu samples. Samples coimplanted with both Ag and Cu are referred to as AgCu samples.

### Physicochemical characterization

#### Morphological analysis

The surfaces of samples were analyzed by scanning electron microscopy using a Zeiss Neon40 field emission scanning electron microscope (Zeiss, Germany). Micrographs were taken at a working distance of 4 mm and a potential of 10 kV using secondary electrons. Energy-dispersive x-ray (EDX) elemental maps were obtained using an EDX detector (INCAPentaFETx3 detector, Oxford Instruments, UK). Two samples of each condition were observed.

#### Surface chemical characterization

The surface composition of the samples and the Auger spectrum were analyzed by x-ray photoelectron spectroscopy (XPS) using an XR50 Mg anode source at 150 W and a Phoibos 150 MCD-9 detector (D8 Advance, SPECS Surface Nano Analysis GmbH, Germany). Spectra were recorded with a pass energy of 25 eV in 0.1-eV steps at a pressure below 7.5 × 10^−9^ mbar. Peak fitting and spectral analysis were performed using the CasaXPS software (version 2.3.16, Casa Software Ltd., UK), and all binding energies were referenced to the C 1s signal (284.8 eV). Two samples were analyzed for each condition.

The concentration as a function of the depth of Ag and Cu atoms implanted into the bulk material was evaluated by XPS depth profiling (PHI VersaProbe II, Physical Electronics), which employs a monochromatic Al Kα source (1,486.6 eV). The voltage and power of the source were kept constant at 10 kV and 24.9 W, respectively. Sputtering was performed with an Ar^+^ ion gun (1 keV) with a diameter of 200 μm, resulting in a sputter rate of 1 nm/min for SiO_2_. After each sputtering cycle, the sputtered area was analyzed. Regions corresponding to Ag 3d and Cu 2p peaks were recorded and converted into atomic concentrations using the PHI MultiPak software (Physical Electronics, USA). In the XPS depth profile graphs, the results were expressed in terms of sputter time rather than depth because there is no direct correlation between the sputter rates of SiO_2_ and Ti6Al4V.

#### Ion release

The release of Ag and Cu ions from the rough samples was assessed following the ISO 10993-5 standard using inductively coupled plasma mass spectrometry (ICP-MS; Agilent 5100 SVDV ICP-OES, CA, USA) for the analyses. Each sample was submerged in 3.5 ml of Hanks’ solution (1 ml per gram of material) and incubated at 37.5 °C for 6 h, 12 h, 18 h, 24 h, 3 d, 7 d, 14 d, and 21 d. One milliliter of the incubated Hanks’ solution was extracted and diluted 1:2 in a 2% nitric acid solution for ICP-MS evaluation. To maintain the initial volume, the extracted solution was replaced with fresh Hanks’ solution. The experiments were conducted in triplicate using 3 samples for each condition. All measurements are reported in parts per billion. The detection limits of the ICP equipment were 0.50 ppb for Ag and 1.00 ppb for Cu.

One-way analysis of variance (ANOVA) was used for multiple comparisons and Tukey’s test was used as a post hoc test.

### Cell assays

#### Cell culture

HCKs (Cell Applications Inc., USA) were maintained in Human Corneal Keratocyte Media (Cell Applications Inc., USA) at 37 °C in a humidified incubator with 5% CO_2_, refreshing the medium every 2 d. Once confluent, the HCKs were detached from the culture flask by incubating with TrypLE (Invitrogen, USA) for 5 min. All experiments were conducted using cells at passages 3 and 4.

#### Immunostaining for cell adhesion and morphology

A minimum of 2 × 10^4^ HCKs were seeded onto rough samples of each condition and incubated in HCK medium without serum for 6 h at 37 °C. For the 24 h time points, after 6 h of incubation, the medium was aspirated and replaced by complete Human Corneal Keratocyte Media. At the selected time points, the cells adhered to the surfaces were fixed for 30 min with 4% (w/v) paraformaldehyde. To assess cell adhesion and morphology, staining of nuclei and actin fibers was performed on both control and implanted samples.

For this analysis, cells were first permeabilized with 500 μl per disk of 0.05% (w/v) Triton X-100 in phosphate-buffered saline (PBS) for 20 min and then blocked with 1% (w/v) bovine serum albumin in PBS for 30 min. Washing between steps was done with PBS-Gly (PBS containing 20 mM glycine) for three 5-min intervals. Next, phalloidin–rhodamine (1:300) was applied in 0.05% (w/v) Triton X-100 in PBS for 1 h in the dark. Cell nuclei were stained with 500 μl per disk of 4′,6-diamidino-2-phenylindole (1:1,000) in PBS-Gly for 2 min in the dark. Next, samples were mounted in Mowiol 4–88, placed on microscope slides, and examined using fluorescence microscopy (Nikon E600, Tokyo, Japan).

Cell number and cell area were quantified using the ImageJ 1.46R software (National Institutes of Health, USA). Cell counts were performed by counting nuclei in 5 fields per disk, and the mean values were calculated. The area of adherent cells was measured for at least 10 cells per field and sample and averaged across samples for each condition. Given that the surfaces used were rough, the real surface area of the samples was measured and used to correct the number of cells per square centimeter and the area of the cells. All cellular studies were conducted in triplicate with 3 samples per condition.

#### Cell proliferation

For proliferation assays, at least 1 × 10^4^ HCKs were plated on each type of rough sample and incubated in serum-free medium for 6 h at 37 °C. After 6 h of incubation, the medium was aspirated and replaced by complete Human Corneal Keratocyte Media. The medium was replaced every 2 d.

At designated time points of 1, 3, 7, and 14 d, the medium was removed, and the samples were washed with PBS before being incubated with 10% (v/v) PrestoBlue (PB) (Invitrogen, USA) to determine the cell number. To calculate the cell number, the fluorescence of the supernatants incubated with the samples was measured at 590 nm. Next, the fluorescence value of the culture medium without cells was subtracted. This adjusted fluorescence value, without the value of the blank, was used to calculate the cell count by interpolating it in a calibration curve of known cell values. Proliferation assays were conducted in triplicate with 3 samples for each condition.

### Microbial assays

#### Microbial culture

The antimicrobial potential of the obtained rough surfaces was evaluated against *P. aeruginosa* and *C. albicans*, 2 common pathogens found in ophthalmic infections [8,29,30]. The reference strains *P. aeruginosa* ATCC 27853 and *C. albicans* ATCC 90028 were used in the adhesion and growth kinetics assays. *C. albicans* was cultured in Sabouraud 2%-dextrose agar plates (Sigma-Aldrich, USA) at 30 °C for 2 to 3 d. *P. aeruginosa* was cultured in Müller–Hinton (MH) broth (Sigma-Aldrich, USA) overnight at 37 °C.

#### Microbial adhesion assays

The overnight culture of *P. aeruginosa* in the stationary phase was centrifuged for 10 min at 1,000×g and then resuspended in sterile PBS (pH 7.4). The bacterial concentration was measured by turbidity at 600 nm, using previously established standards as a reference. The bacterial suspension was adjusted to the optimal density (1 × 10^5^ colony-forming units [CFU]/ml) in MH broth. For *C. albicans*, 4 to 8 colonies of the solid culture were resuspended in sterile PBS, and the fungal concentration was measured by turbidity at 600 nm, using previously established standards as a reference.

The fungal suspension was adjusted to the optimal density (2 × 10^4^ CFU/ml) in Roswell Park Memorial Institute (RPMI) broth.

Prior to the adhesion assays, the samples were sterilized by treatment with 70% ethanol for 30 min and then thoroughly rinsed with sterile PBS to remove any trace of ethanol. The samples from the different conditions were placed in a 24-well plate and incubated with 1 ml of the adjusted microbial suspension for 2 h at 37 °C (*P. aeruginosa*) or 30 °C (*C. albicans*).

#### Microbial survival after release killing

After incubation, the medium containing planktonic microorganisms was aspirated, and the samples were rinsed 3 times with sterile PBS. The supernatants containing nonadherent microorganisms were serially diluted and seeded on MH agar for *P. aeruginosa* and Sabouraud plates for *C. albicans*. The plates were incubated at 37 °C or at 30 °C for 24 h.

After incubation, the resulting colonies from surviving CFUs were counted to determine the number of viable microorganisms (CFU/ml) remaining in the supernatant. All experiments were performed in triplicate for each type of sample.

The results were normalized to the control samples (SB samples) to express the relative number of surviving microorganisms.

#### Microbial survival after contact killing

The contaminated disks from the adhesion assay were transferred to sterile tubes containing 1 ml of 50% MH in sterile PBS (MH–PBS) for assays with *P. aeruginosa* or 1 ml of RPMI for *C. albicans*. Adherent microorganisms were detached by vortexing for 15 min. To ensure effective dislodging of microorganisms from the surfaces, after the first vortexing step, the disks were transferred to new sterile tubes containing 1 ml of MH–PBS or RPMI and vortexed again for 15 min. Microbial suspensions from each vortexing step were then serially diluted in MH–PBS and seeded on MH agar plates for *P. aeruginosa*. For *C. albicans*, the process was the same, but the dilutions were made with PBS, and Sabouraud plates were used for seeding. The plates were incubated at 37 °C or for 30 °C 24 h, and the surviving CFUs were counted. All experiments were performed in triplicate for each type of sample. The results were normalized to SB samples.

#### Microbial adhesion and morphology by FESEM

The same process mentioned in the adhesion test section was followed. After the 2-h adhesion assay, the medium with the nonadherent microorganisms was discarded, and the samples were washed 3 times with PBS. Subsequently, the adhered microbes were fixed to the surfaces using 2.5% glutaraldehyde in PBS at 4 °C for 15 min. Then, the samples were prepared for field emission scanning electron microscopy (FESEM). Samples were dehydrated with a series of ethanol solutions (30%, 50%, 70%, 90%, 95%, and 100%), each step lasting 15 min. Next, the samples were coated with carbon and observed with FESEM. Electronic images were taken at a working distance of 10 mm and a potential of 5 kV using secondary electrons. Three samples of each condition were observed.

#### Microbial growth kinetics

The samples from all conditions were placed into 48-well plates and immersed in 1 ml of microbial suspension for 2 h to allow microbial adhesion to the tested surfaces. For *P. aeruginosa*, a suspension with an approximate density of 1 × 10^5^ CFU/ml in MH was used.

Meanwhile, for *C. albicans*, a suspension with an approximate density of 2 × 10^4^ CFU/ml in RPMI was used. After the 2-h adhesion period, the medium containing planktonic microorganisms was discarded, and the disks with the adhered microorganisms were washed with sterile PBS. The titanium disks with adhered microorganisms were then incubated at 37 °C for 22 h in fresh MH or RPMI medium containing 10% (v/v) PB metabolic dye. The kinetics of microbial growth on the adhered microorganisms were measured fluorometrically in accordance with the instructions provided by the manufacturer of PB, utilizing a multimode plate reader (EnSpire 2300, PerkinElmer, Waltham, MA, USA) at the set time points until the endpoint of 22 h.

### Co-culture assays

These assays were conducted following the preimplantation infection method. First, the rough samples were infected according to the steps described in the microbial adhesion section. Following the 2-h incubation, the medium was removed, and the samples were washed 3 times with sterile PBS.

Subsequently, HCK cells, freshly resuspended in serum-free medium without penicillin and streptomycin and supplemented with 2% MH broth (for *P. aeruginosa*) or 2% RPMI (for *C. albicans*), were seeded on microbially covered surfaces at a density of 2 × 10^4^ cells/disk. Microorganisms and HCK cells were incubated at 37 °C in a humidified atmosphere with 5% CO_2_ for 6 or 24 h. At the time points, the medium was discarded, the samples were rinsed with PBS, and the cells were fixed in 4% paraformaldehyde.

#### Immunostaining

The fixed samples were stained as mentioned previously to visualize cell nuclei and actin filaments. The samples were examined by confocal laser scanning microscopy using a Leica TCS SP8 X microscope (Leica Microsystems GmbH, Germany).

Images were analyzed with the ImageJ 1.51w software (National Institutes of Health, USA) to determine the HCK area and surface coverage. All experiments were performed in duplicate for each surface type. The area of adherent HCKs was measured for at least 10 cells per field and averaged across the replicates for each condition. The value of the area covered by HCKs relative to the area of the imaged fields was used to calculate cell surface coverage using Eq. 1, where A represents each condition used in the co-culture experiments and therefore infected with microorganisms. Given that the surfaces used were rough, the actual surface area of the samples was measured and used to correct the cell area.

$$\begin{array}{c} \begin{array}{c} \begin{array}{c} \mathrm{HCK}\;\text{surface coverage}\;\left(\%\right)=\\ \left(\text{area covered}\;\mathrm{by}\;\text{HCKs in condition}\;A\right)/\left(\text{total surface area}\right)*100 \end{array} \end{array} \end{array}$$ (1)

Subsequently, the surface coverage values were normalized to the SB samples without microorganisms, as depicted in Eq. 2. The values were calculated for each replicate of each condition, and the mean and standard deviation for each condition were then calculated.

$$\begin{array}{c} \begin{array}{c} \begin{array}{c} \mathrm{HCK}\;\text{surface coverage}\;\left(\%\;\mathrm{vs}.\mathrm{SB}\right)=\\ \left(\text{surface coverage in condition}\;A\;\left(\%\right)\right)/\left(\text{surface coverage in}\;\mathrm{SB}\;\left(\%\right)\right)*100 \end{array} \end{array} \end{array}$$ (2)

#### Field emission scanning electron microscopy

The fixed co-culture samples were dehydrated with a series of ethanol solutions (30%, 50%, 70%, 90%, 95%, and 100%), each step lasting 15 min. Next, the samples were coated with platinum/palladium and observed with FESEM.

Electronic images were taken at a working distance of 10 mm and a potential of 5 kV using secondary electrons. Two samples of each condition were observed.

### Statistical analysis

In this study, the data are reported as mean value ± standard deviation for each condition. The Minitab 19 software (Minitab, USA) was used to assess statistically significant differences between groups. Prior to analysis, sample normality was verified. Depending on the results, either an ANOVA test or a nonparametric Kruskal–Wallis’s test was performed to determine the *P* value.

Statistical significance was set at *P* < 0.05, with a 95% confidence interval used unless otherwise specified. In Results, conditions with statistically significant differences are indicated by the same symbol.

## Results

### Physicochemical characterization

#### Surface topography

**Fig. 2.**
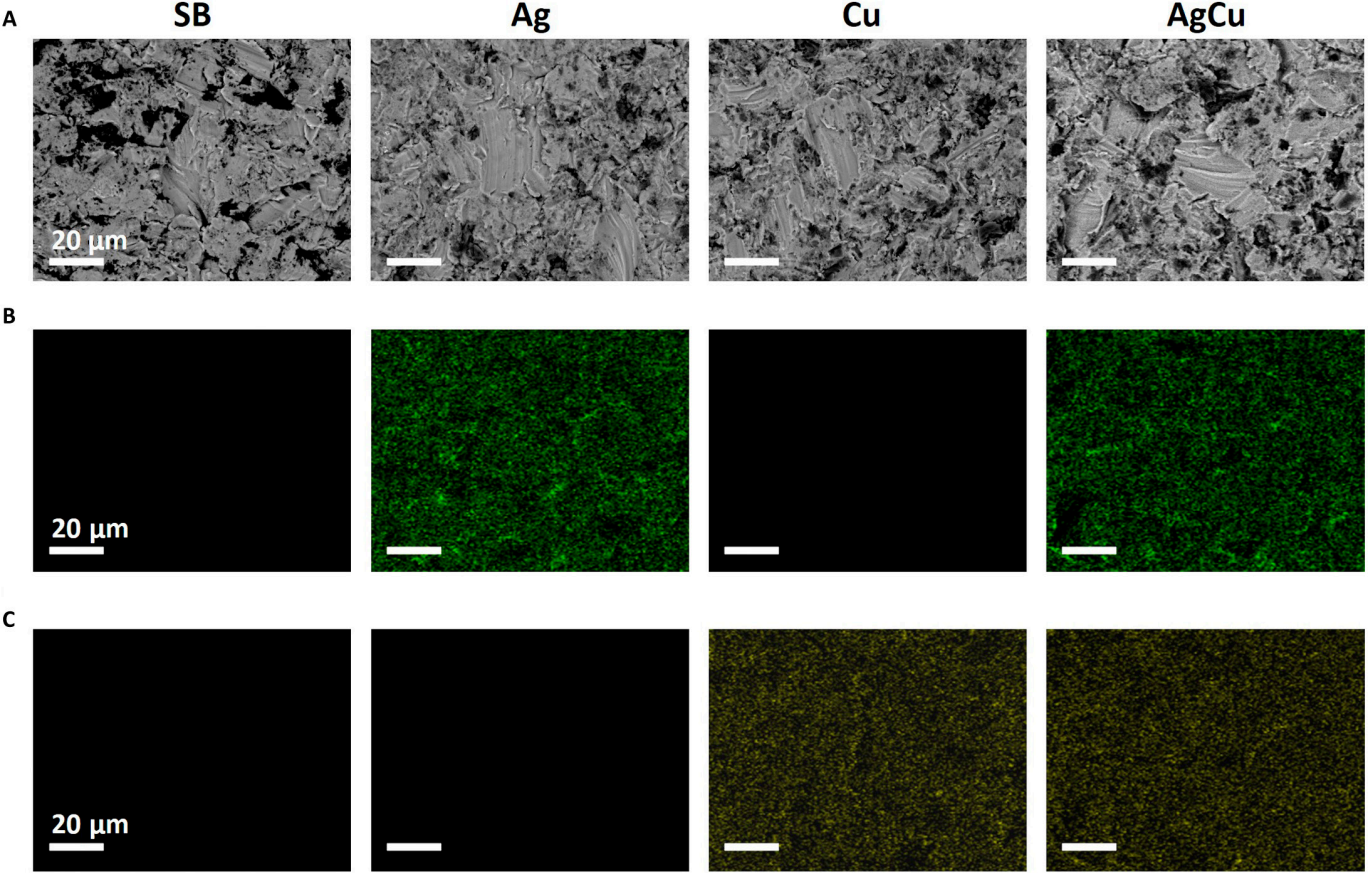
(A) FESEM images of rough Ti6Al4V samples (SB), Ti samples monoimplanted with silver (Ag), Ti samples monoimplanted with copper (Cu), and Ti samples coimplanted with silver and copper (AgCu). (B) Energy-dispersive x-ray (EDX) color mapping of Ag atoms in SB, Ag, Cu, and AgCu samples. (C) EDX color mapping of Cu atoms in SB, Ag, Cu, and AgCu samples. Scale bars = 20 μm.

To study changes in composition and topography after ion implantation, the rough samples were observed through FESEM and EDX (Fig. 2).

FESEM micrographs revealed an irregular topography, characteristic of the sandblasting process. The topography of the different conditions was very similar, and no precipitates were observed after the ion implantation process (Fig. 2A). The EDX analysis confirmed the presence of Ag and Cu atoms in the implanted surfaces (Fig. 2B and C).

#### XPS studies

**Fig. 3.**
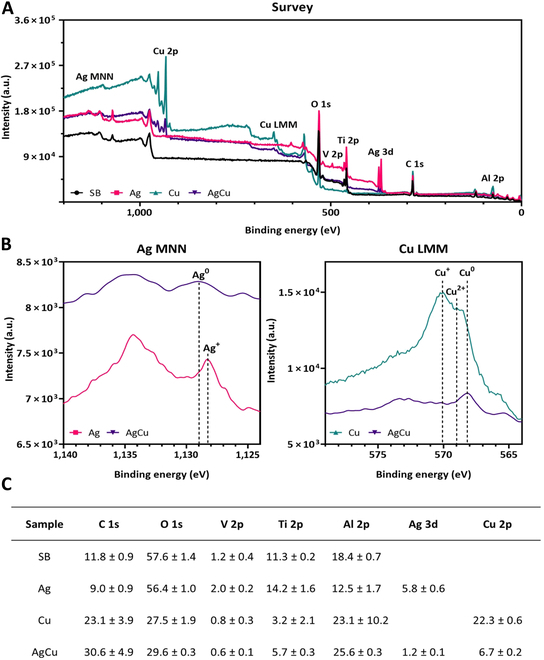
(A) XPS survey spectra of rough Ti6Al4V samples (SB) (black line), Ti samples monoimplanted with silver (Ag) (pink line), Ti samples monoimplanted with copper (Cu) (green line), and Ti samples coimplanted with silver and copper (AgCu) (purple line). (B) Auger spectra of the Ag MNN and Cu LMN regions of samples Ag, Cu, and AgCu. (C) Chemical composition (atomic percentage) measured by XPS (mean ± standard deviation) in the samples of SB, Ag, Cu, and AgCu conditions.

After verifying the incorporation of Ag and Cu atoms, a more detailed characterization was carried out using XPS. The survey spectra of all samples and the Auger spectra of the ion-implanted samples were acquired. The high-resolution spectra of the C 1s, O 1s, V 2p, Al 2p, Ag 3d, Cu 2p, and Ti 2p signals were used to quantify the surface atomic percentages (Fig. 3).

In the survey spectra, the Ag 3d, Cu 2p, Ag MNN, and Cu LMN regions were detected only in the implanted samples as expected (Fig. 3A).

The quantification of atomic percentages on the surfaces revealed the presence of a passive layer and the incorporation of Ag and Cu in the respective implanted samples. Ag 3d and Cu 2p signals increased in the implanted samples. The atomic concentration of Ag and Cu was higher in the monoimplanted samples. Additionally, after ion implantation, there was an increase in the C 1s and Al 2p signals, except in the Ag monoimplanted samples (Fig. 3C).

**Fig. 4.**
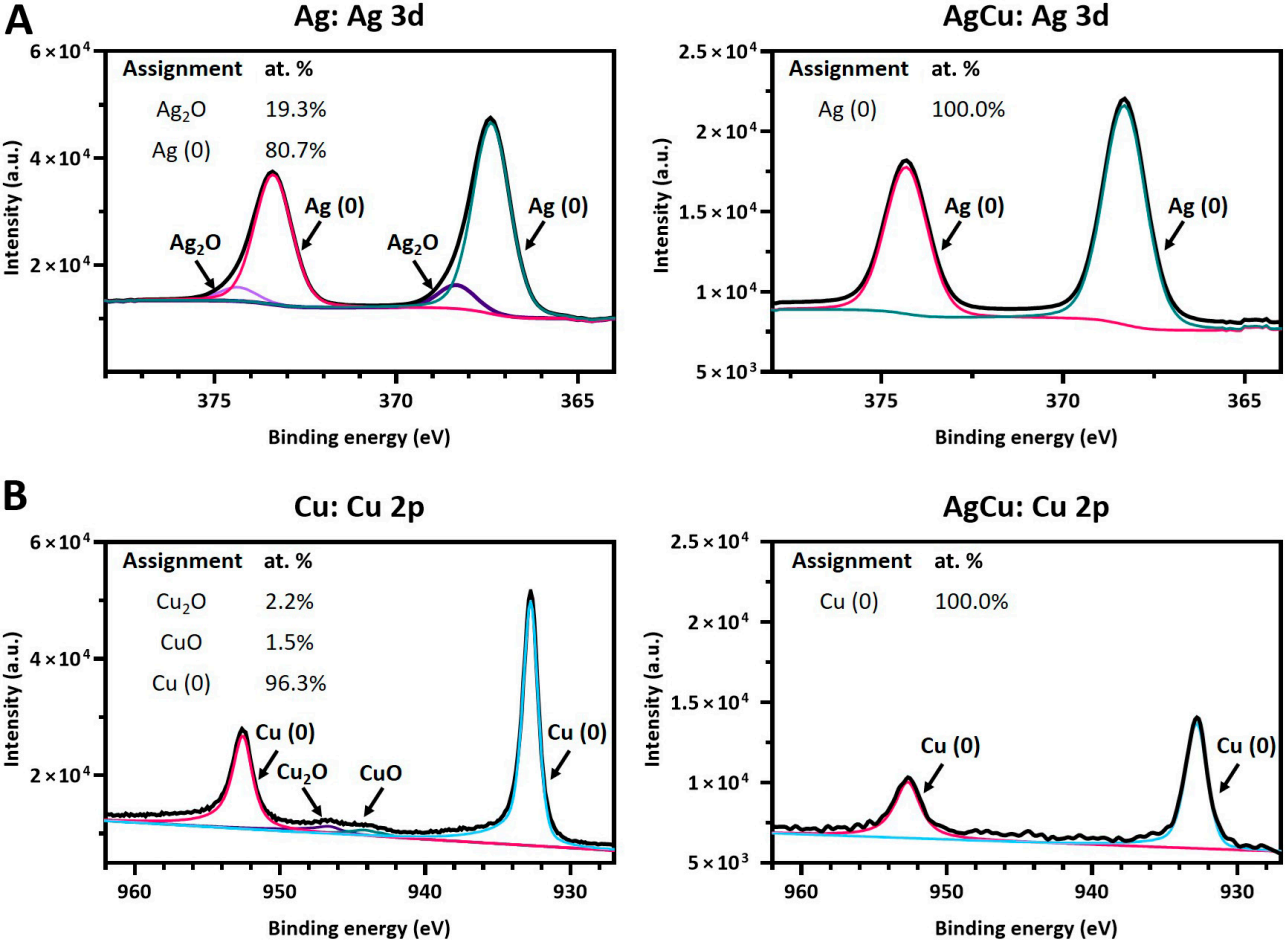
(A) Deconvoluted high-resolution Ag 3d core-level spectra of Ti samples monoimplanted with silver (Ag) and Ti samples coimplanted with silver and copper (AgCu). (B) Decomposed high-resolution Cu 2p core-level spectra of Ti samples monoimplanted with copper (Cu) and Ti samples coimplanted with silver and copper (AgCu) samples.

The Ag MNN Auger spectrum revealed that silver was in metallic state in coimplanted samples, whereas it appeared as a mixture of metallic silver and oxide form Ag_2_O in the Ag monoimplanted samples. Similarly, the Auger spectrum for the Cu LMM region showed a combination of copper oxides (CuO and Cu_2_O) and metallic copper in the Cu monoimplanted samples, while only the characteristic spectrum of metallic copper was observed in the coimplanted samples (Fig. 3B). Since Auger spectroscopy does not provide quantitative information, high-resolution spectra of the Ag 3d and Cu 2p regions were analyzed to determine the bonding states of Ag and Cu and their relative proportions in the implanted samples (Fig. 4).

The high-resolution spectrum of Ag 3d revealed the presence of 2 peaks in all conditions: Ag 3d_5/2_ and Ag 3d_3/2_. For the Ag monoimplanted samples, the Ag 3d_5/2_ peak could be deconvoluted into 2 components, corresponding to metallic Ag (367.4 eV) and oxide Ag_2_O (367.5 eV). The Ag 3d_3/2_ peak also consisted of contributions from both metallic Ag and oxide Ag_2_O according to the literature [31,32]. The ratio Ag/Ag oxides was 4.2, indicating prevalence of metallic Ag. In the case of coimplanted samples, the Ag 3d_5/2_ and Ag 3d_3/2_ peaks were separated by 6 eV and consisted of a single contribution from metallic Ag (Fig. 4A).

The high-resolution spectrum of Cu 2p revealed 2 main peaks under all conditions: Cu 2p_1/2_ and Cu 2p_3/2_. For Cu monoimplanted samples, the Cu 2p_1/2_ (932.7 eV) and Cu 2p_3/2_ (952.5 eV) peaks were assigned to metallic Cu. Two small satellite peaks were also observed, corresponding to CuO (943.1 eV) and Cu_2_O (946.6 eV), in agreement with the literature [33,34]. The ratio Cu/Cu oxides was 25.5, indicating prevalence of metallic Cu. In the coimplanted samples, the Cu 2p doublet peaks were separated by 19.9 eV, with a single contribution from metallic Cu (Fig. 4B).

**Fig. 5.**
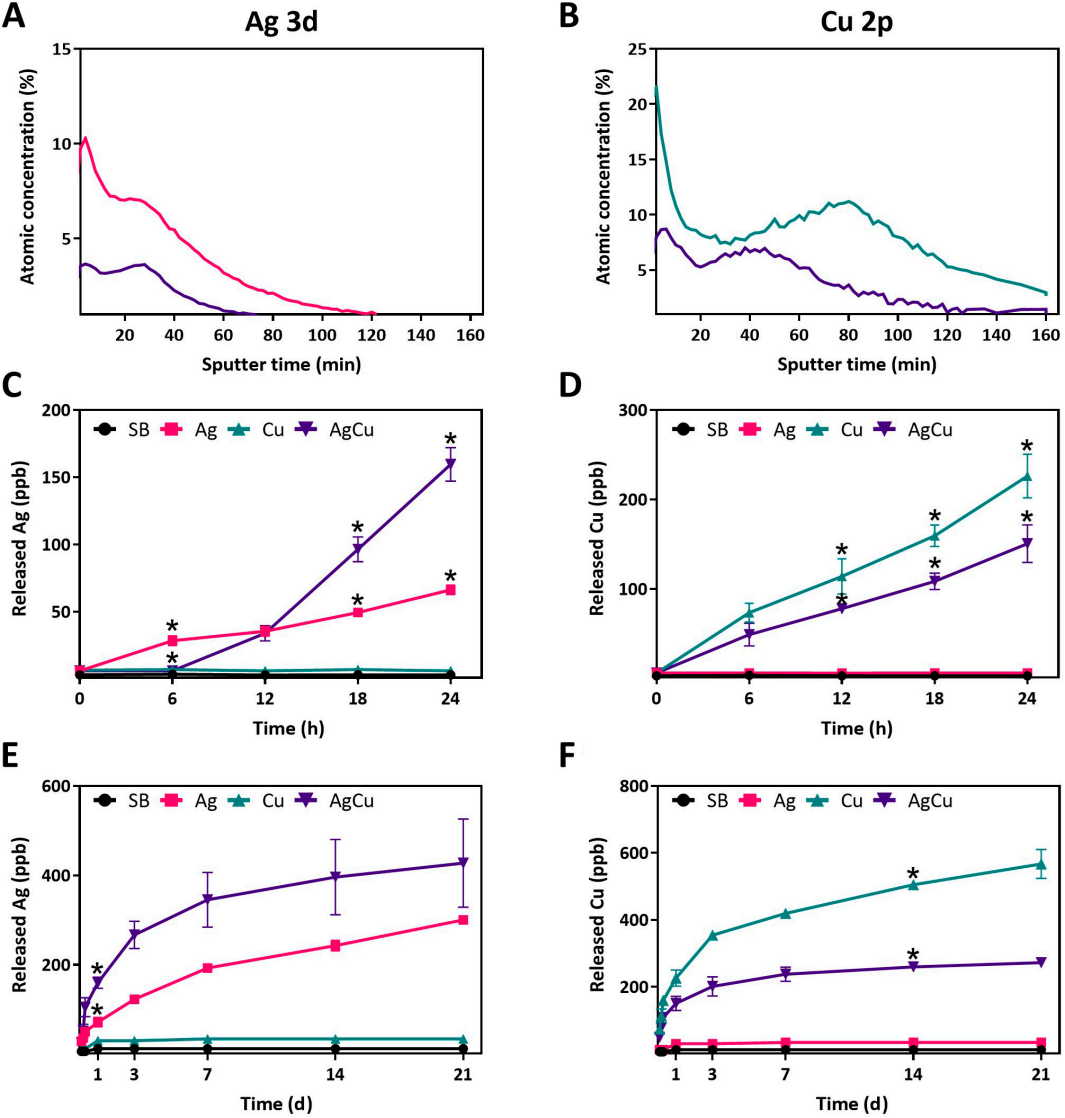
(A) XPS depth profile atomic concentrations of Ag in rough Ti samples monoimplanted with silver (Ag) and Ti samples coimplanted with silver and copper (AgCu). (B) XPS depth profile atomic concentrations of Cu in Cu and AgCu samples. Ion release profiles analyzed using inductively coupled plasma mass spectrometry (ICP-MS) of (C) Ag released ions (ppb) in SB, Ag, Cu, and AgCu samples the first 24 h; (D) Cu released ions (ppb) in SB, Ag, Cu, and AgCu samples the first 24 h; (E) Ag released ions (ppb) in SB, Ag, Cu, and AgCu samples; and (F) Cu released ions (ppb) in SB, Ag, Cu, and AgCu samples (*P < 0.05, Tukey’s test).

After characterizing the surface composition, XPS depth profiling was performed to analyze how the atomic percentages of Ag and Cu change with depth (Fig. 5).

The XPS depth profile of Ag% showed that the maximum concentrations on the surface and the bulk of the material were 7.50% and 10.3% for Ag monoimplanted samples, respectively, and 2.47% and 3.66% for AgCu samples, respectively. After 160 min of sputtering, the Ag concentration was almost 0% in Ag monoimplanted samples, while this value was reached after 60 min in AgCu samples (Fig. 5A). The profile of Cu% showed that the Cu concentration was higher in the monoimplanted Cu samples, with surface and maximum bulk values of 20.0% and 21.54%, respectively, and 2.66% remaining after 160 min. In contrast, the AgCu coimplanted samples showed surface and bulk values of 5.50% and 8.70%, with 1.00% remaining after 160 min (Fig. 5B).

These results demonstrate that the highest metal concentrations were observed at specific depths within the bulk material. However, establishing a reliable relationship between sputter time and depth was challenging because, despite the sputtering rate in the SiO_2_ pattern being known, and consequently the depth, it does not directly correspond to the sputtering rate in Ti6Al4V. For this reason, the results are presented in terms of sputtering time rather than depth.

Cu ions were incorporated in higher amounts and to greater depths than Ag, due to the lower atomic mass of Cu ions. The simultaneous implantation of both ions resulted in reduced concentrations and shallower depths for each ion. Additionally, Cu and Ag exhibited 2 peaks of maximum concentration, attributable to the presence of variously charged copper and silver ions (Cu^+^ and Cu^2+^ and Ag^+^ and Ag^2+^) in the plasma generated by the cathodic arc discharge.

#### Ion release

The confirmed penetration of Ag and Cu ions into the deeper layers of the bulk material raised the question of whether these ions, including those in surface, could be released over time. To investigate this, ion release assays were conducted over a period of 21 d and the release ions were measured using ICP-MS, as shown in Fig. 5.

The analysis of Ag ion release showed values in the first 24 h ranging from 28.5 to 66.3 ppb in Ag monoimplanted samples and from 34 to 159.5 ppb in AgCu coimplanted samples. After 21 d, the values increased to final concentrations of 300.4 ppb for Ag monoimplanted samples and 427.5 ppb for AgCu coimplanted samples. The release of Ag ions began earlier in the Ag monoimplanted samples, starting from 6 h, whereas in the AgCu samples, the release started after the first 12 h. Surprisingly, after 24 h, Ag ion release was higher in the AgCu condition, reaching a final value greater than that in the Ag monoimplanted condition. However, there were no statistically significant differences when comparing the final Ag ion release values after 24 h between the Ag and AgCu conditions. Similarly, no statistically significant differences were observed when comparing the values on days 7, 14, and 21 for the AgCu samples, while there were differences for the Ag samples, leading to the conclusion that the AgCu samples had reached a plateau. This could be explained by the higher initial surface concentration in Ag monoimplanted samples, which facilitated the initial release. In contrast, in AgCu conditions, the ions from the bulk were closer to the surface, facilitating their release once the surface ions were depleted, resulting in a higher overall concentration. After 21 d, Ag ion release reached a plateau in the AgCu condition but continued to increase in the Ag condition (Fig. 5C and E).

Similarly, the analysis of Cu ion release showed values in the first 24 h ranging from 73.5 to 232.6 ppb in Cu monoimplanted samples and from 49 to 150.6 ppb in AgCu samples. The release of Cu ions started after 6 h in both conditions. The final values were 567.0 ppb in Cu monoimplanted samples and 272.3 ppb in AgCu samples.

There were no statistically significant differences in the final Cu ion release values after 24 h between the Cu and AgCu conditions. Additionally, the comparison of the values on days 7, 14, and 21 for the AgCu samples was not statistically significant, demonstrating that a plateau in ion release had been reached for the AgCu samples. In the Cu samples, there was significance between days 7 and 14, but not between days 14 and 21 (Fig. 5D and F). As with Ag ions, the increased release in Cu monoimplanted samples was attributed to a greater concentration of Cu at the surface of the samples. The sustained ion release in monoimplanted samples was attributed to the enhanced concentration and depth of ions in the bulk, which generates a slower but prolonged release over time.

### Biological characterization

#### Cell adhesion and proliferation

**Fig. 6.**
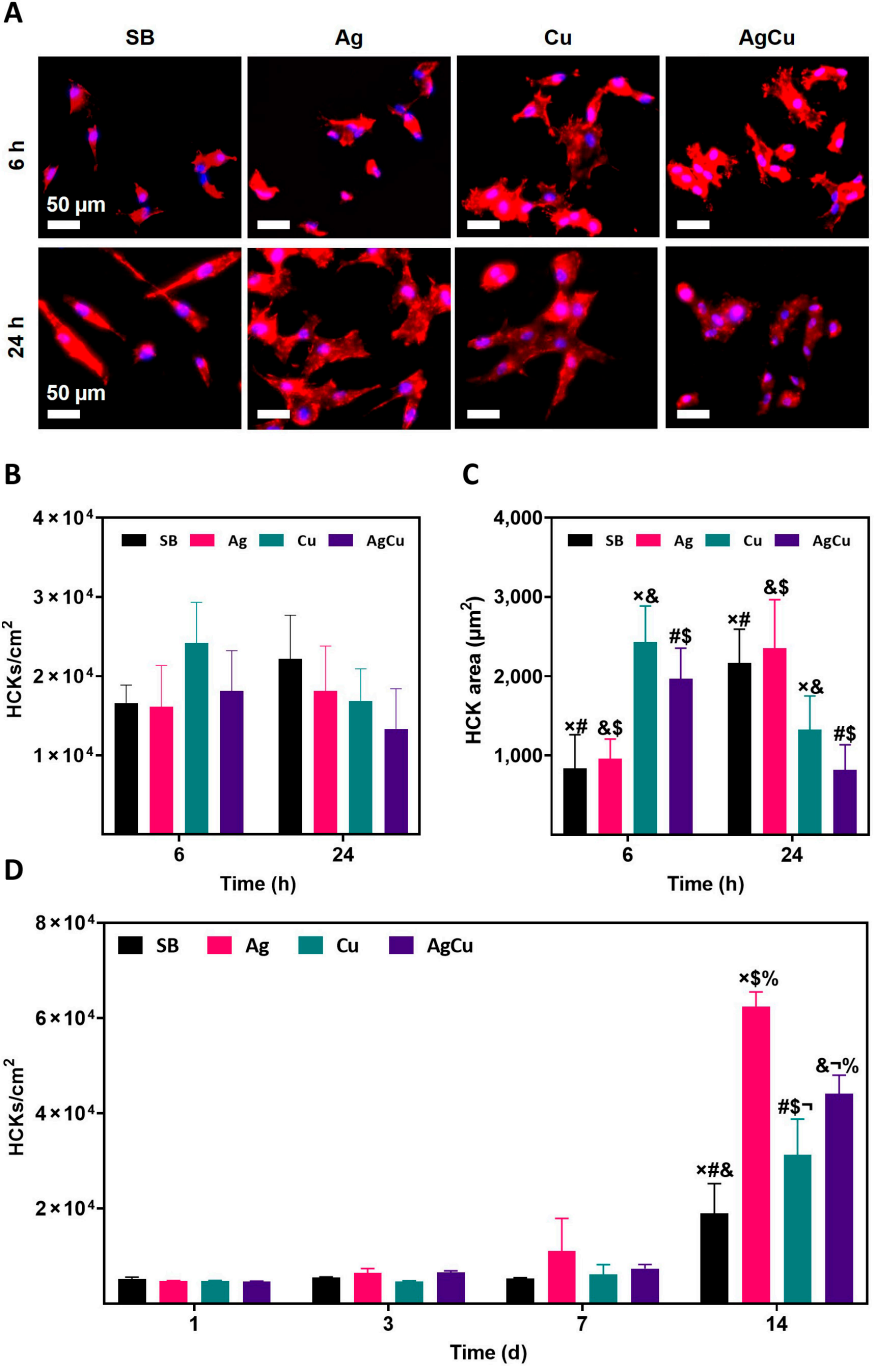
(A) Cell adhesion assays with HCKs at 6 and 24 h in samples of Ti (SB), Ti samples monoimplanted with silver (Ag), Ti samples monoimplanted with copper (Cu), and Ti samples coimplanted with silver and copper (AgCu). F-actin and nucleus immunostaining (scale bar = 50 μm). (B) Cell quantification. (C) Cell area quantification. (D) HCK proliferation after 1, 3, 7, and 14 d of culture. Results are presented as mean ± standard deviation, and conditions with statistically significant differences (*P* ≤ 0.05) are indicated by the same symbol.

Following the characterization of the physicochemical properties, the potential adverse effects of the implanted ions on human HCKs were assessed. Adhesion assays were conducted at 6 and 24 h to evaluate the morphology, number of adhered HCKs, and HCKs’ area. Additionally, the long-term effects on HCKs were investigated with proliferation studies (Fig. 6).

Immunohistochemistry images revealed that HCKs adhered well and were evenly distributed on all surfaces at both time points (Fig. 6A). Cell quantification showed no significant differences in cell numbers at 6 h across all conditions. However, after 24 h, a slight decrease in cell number was observed in conditions with Cu implantation (Fig. 6B). Initially, cells in Cu samples exhibited a more spread-out phenotype with larger areas, but by 24 h, cells across all conditions were more spread, indicating good adhesion (Fig. 6C). Proliferation assays demonstrated an increase in cell population over time in Ag and AgCu conditions, while growth in SB and Cu conditions was slower, with a minimal increase until day 14 (Fig. 6D). These results suggest that the implanted ions do not adversely affect long-term cell proliferation.

#### Antibacterial studies

**Fig. 7.**
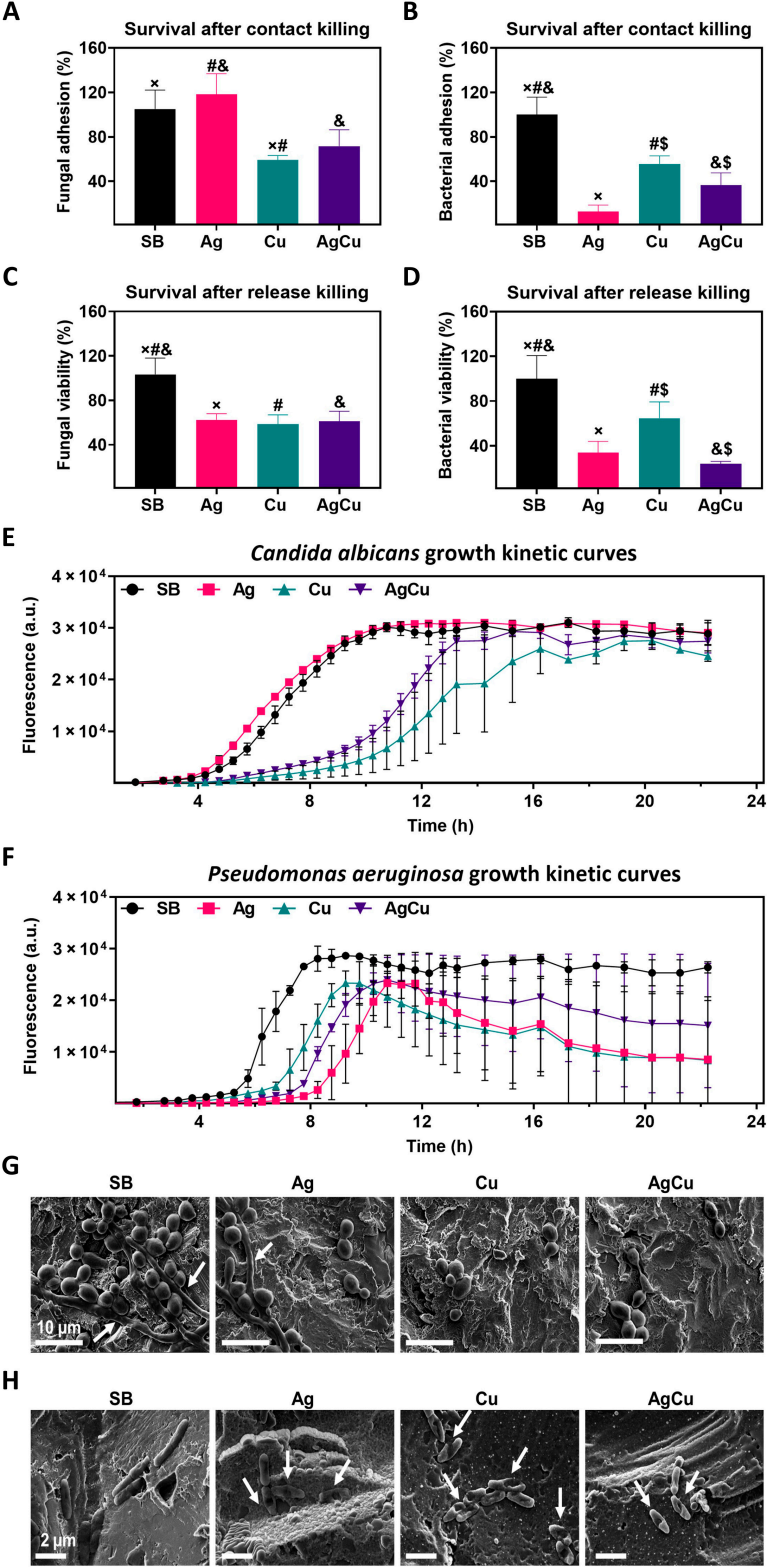
(A) Two-hour direct adhesion colony-forming units (CFUs) of *Candida albicans* in samples of Ti (SB), Ti samples monoimplanted with silver (Ag), Ti samples monoimplanted with copper (Cu), and Ti samples coimplanted with silver and copper (AgCu). The percentage of fungal viability referred to the SB control. (B) Two-hour direct adhesion CFUs of *Pseudomonas aeruginosa* in SB, Ag, Cu, and AgCu samples. The percentage of bacterial viability referred to the SB control. (C) CFUs in the supernatants of SB, Ag, Cu, and AgCu samples after 2-h adhesion assays with *C. albicans*. The percentage of fungal viability referred to the SB control. (D) CFUs in the supernatants of SB, Ag, Cu, and AgCu samples after 2-h adhesion assays with *P. aeruginosa*. The percentage of bacterial viability referred to the SB control. Results are presented as mean ± standard deviation, and conditions with statistically significant differences (*P* ≤ 0.05) are indicated by the same symbol. (E) Growth kinetic curves of *C. albicans* through 22 h, after 2-h adhesion assays with SB, Ag, Cu, and AgCu samples. (F) Growth kinetic curves of *P. aeruginosa* through 22 h, after 2-h adhesion assays with SB, Ag, Cu, and AgCu samples. (G) FESEM images after 2-h adhesion of *C. albicans* in SB, Ag, Cu, and AgCu samples. Scale bars = 10 μm. Arrows indicate pseudohyphae. (H) FESEM images after 2-h adhesion of *P. aeruginosa* in SB, Ag, Cu, and AgCu samples. Scale bars = 2 μm. Arrows indicate bacteria with damaged membranes.

Following the confirmation of non-harmful effects on HCKs, the antimicrobial efficacy of the ion-implanted samples was assessed. Microbial adhesion assays were conducted at 2 h to test early antimicrobial activity against *P. aeruginosa* and *C. albicans*. This was evaluated by counting CFUs from both microorganisms that adhered directly to the samples (Fig. 7A and B) and those present in the supernatants (Fig. 7C and D). To investigate the sustained antimicrobial effects, kinetic growth assays were performed over 22 h (Fig. 7E and F). Additionally, FESEM was used to observe any morphological changes in the microorganisms after exposure to the samples (Fig. 7G and H).

The CFUs of *C. albicans* recovered from the Cu-implanted conditions were significantly fewer than those from SB and Ag conditions, suggesting that Cu primarily kills the fungi upon contact or inhibits their adhesion to the Ti surfaces (Fig. 7A). This aligns with the understanding that Cu plays a more crucial role in fungal inhibition, starting to affect adhesion early on. In contrast, *P. aeruginosa* adhesion was more effectively reduced by Ag in the early hours, likely due to the more superficial placement of Ag ions and their earlier release, as shown by XPS depth profiles and ICP results (Fig. 5A and B).

This suggests that Ag exerts a stronger bactericidal effect in the short term, compared to Cu (Fig. 7B). Plating CFUs from the supernatants showed a significant reduction in the viability of *C. albicans* in all ion-implanted conditions, confirming that both Ag and Cu ions decrease fungal viability (Fig. 7C).

However, Cu showed a slower release compared to Ag, resulting in similar inhibitory effects on fungal release killing at early hours (Fig. 7C). For *P. aeruginosa*, the number of CFUs was markedly lower in both Ag- and Cu-implanted surfaces, confirming the bactericidal effects of these ions. Again, Ag showed a stronger effect at earlier time points due to its faster release (Fig. 7D).

Growth kinetic assays revealed that surviving *C. albicans* from SB and Ag samples entered the exponential growth phase between 6 and 12 h.

In contrast, fungi from AgCu conditions experienced a 1.5- to 2-h delay in growth, and those from Cu conditions showed a 3-h delay. These results indicate that fungal growth is highly sensitive to the presence of Cu, which significantly delays *C. albicans* growth. In comparison, Ag showed minimal interference with fungal growth. After 22 h, fungal populations in Cu and AgCu samples were slightly inferior those in the SB condition (Fig. 7E). For *P. aeruginosa*, growth began at 6 h post-adhesion in SB samples, with an exponential phase between 6 and 8 h. Bacteria from Cu and AgCu conditions exhibited a 2-h delay, while those from Ag samples experienced a 4-h delay, indicating that bacterial growth is highly sensitive to both Cu and Ag ions. After 24 h, ion-implanted samples showed a reduction in viable bacteria starting around 12 h, highlighting the bactericidal effects of Cu and Ag. The stabilization of the microbial population is often linked to biofilm development, marked by slowed proliferation [35]. In this case, the continued release of Cu and Ag ions could inhibit biofilm formation, as evidenced by the reduced bacterial growth after 12 h (Fig. 7F). The low levels of Cu ion release, as well as the release of surface Cu ions, lead to a reduction in the initial population of *C. albicans* by inhibiting growth during the first few hours. However, this is not sufficient to achieve a significant reduction after 22 h.

FESEM analysis revealed more *C. albicans* in yeast form and the presence of some pseudohyphae in SB and Ag conditions. The formation of pseudohyphae increases resistance to the immune system and is associated with greater pathogenicity [36]. Therefore, the antifungal effect of Cu was verified due to the reduction in fungal presence and fewer pseudohyphae in Cu-implanted samples, reinforcing the stronger antifungal effect of Cu compared to Ag (Fig. 7G). For *P. aeruginosa*, cell wall damage was evident across all ion-implanted samples (Fig. 7H). However, based on the bacterial adhesion results (Fig. 7B), Ag presence reduced bacterial adhesion more effectively than Cu at early exposure hours.

#### Co-culture studies

##### Six-hour co-culture

**Fig. 8.**
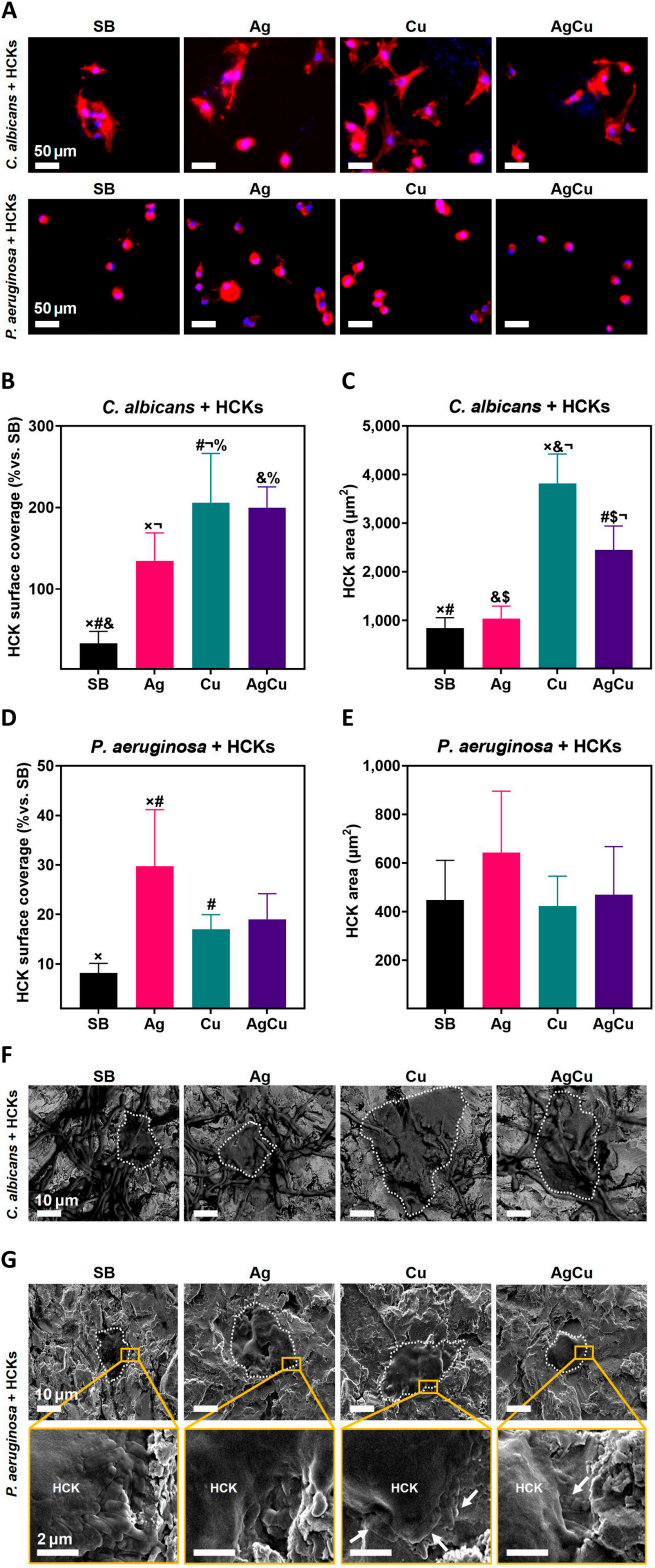
Six-hour co-culture in the preimplantation infection method of HCKs with *P. aeruginosa* or *C. albicans* in samples of Ti (SB), Ti samples monoimplanted with silver (Ag), Ti samples monoimplanted with copper (Cu), and Ti samples coimplanted with silver and copper (AgCu). (A) Immunostaining images showing F-actin and nucleus (scale bar = 50 μm). (B) Surface coverage (%) referring to not contaminated SB samples in co-culture with *C. albicans*. (C) HCK area quantification in co-culture with *C. albicans*. (D) Surface coverage (%) referring to not contaminated SB samples in co-culture with *P. aeruginosa*. (E) HCK area quantification in co-culture with *P. aeruginosa*. Results are presented as mean ± standard deviation, and conditions with statistically significant differences (*P* ≤ 0.05) are indicated by the same symbol. (F) FESEM images of 6-h co-culture of HCKs with *C. albicans* in SB, Ag, Cu, and AgCu samples. Scale bars = 10 μm. Cell contour is defined by dashed lines. (G) FESEM images of 6-h co-culture of HCKs with *P. aeruginosa* in SB, Ag, Cu, and AgCu samples. Scale bars = 2 μm. Arrows indicate bacteria surrounding HCKs.

**Fig. 9.**
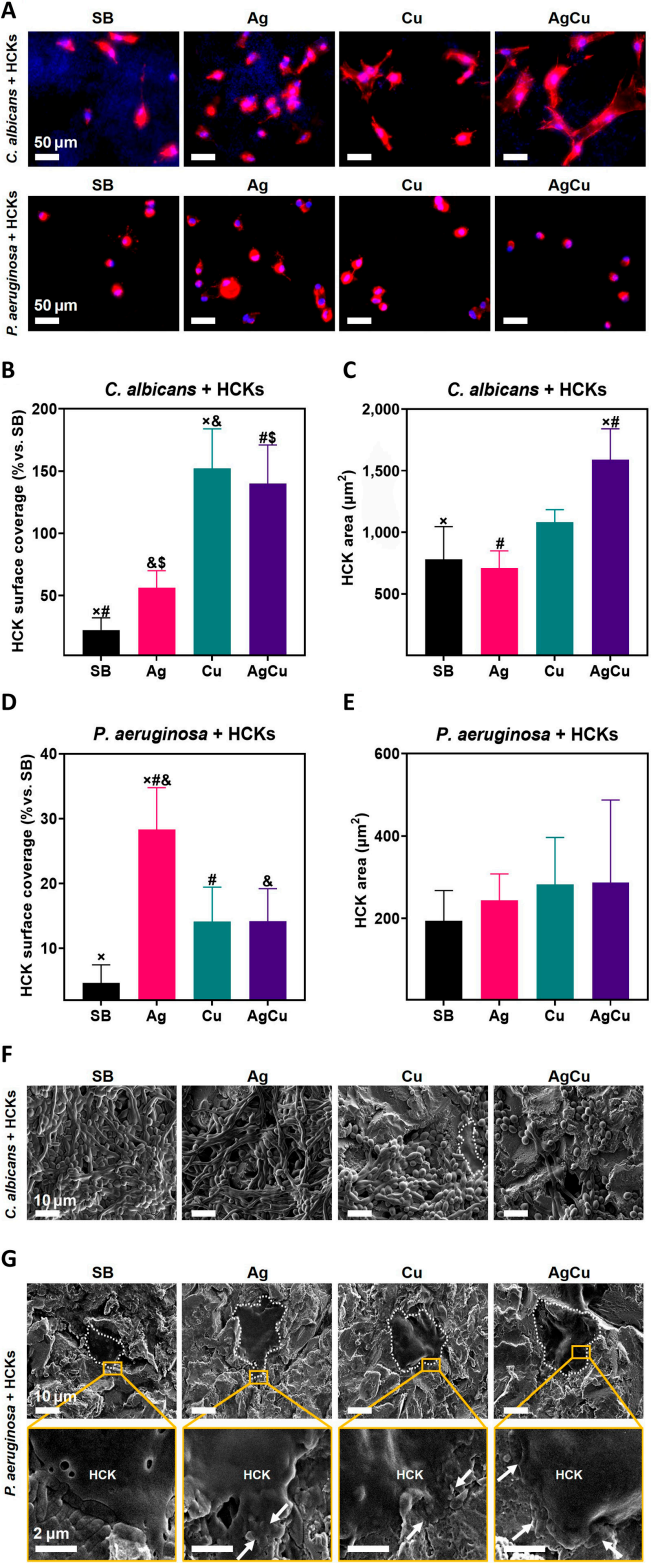
Twenty-four-hour co-culture in the preimplantation infection method of HCKs with *P. aeruginosa* or *C. albicans* in samples of Ti (SB), Ti samples monoimplanted with silver (Ag), Ti samples monoimplanted with copper (Cu), and Ti samples coimplanted with silver and copper (AgCu). (A) Immunostaining images showing F-actin and nucleus (scale bar = 50 μm). (B) Surface coverage (%) referring to not contaminated SB samples in co-culture with *C. albicans*. (C) HCK area quantification in co-culture with *C. albicans*. (D) Surface coverage (%) referring to not contaminated SB samples in co-culture with *P. aeruginosa*. (E) HCK area quantification in co-culture with *P. aeruginosa*. Results are presented as mean ± standard deviation, and conditions with statistically significant differences (*P* ≤ 0.05) are indicated by the same symbol. (F) FESEM images of 24-h co-culture of HCKs with *C. albicans* in SB, Ag, Cu, and AgCu samples. Scale bars = 10 μm. Cell contour is defined by dashed lines. (G) FESEM images of 24-h co-culture of HCKs with *P. aeruginosa* in SB, Ag, Cu, and AgCu samples. Scale bars = 2 μm. Arrows indicate bacteria surrounding HCKs.

After confirming the antimicrobial properties and HCK compatibility of the ion-implanted surfaces, their performance was assessed in a “race for the surface” in a preimplantation infection approach. Co-culture assays were conducted with HCKs and either *P. aeruginosa* or *C. albicans*. To simulate an infection scenario, samples were contaminated with the microorganisms for 2 h before seeding HCKs and then incubated for an additional 6 or 24 h. At selected time points, the Ti disks were stained to evaluate HCKs’ surface coverage compared to that of pathogen-free Ti controls, as well as the HCKs’ area. FESEM analysis was performed to observe morphological changes and the distribution of HCKs and microorganisms on the surface of the samples (Figs. 8 and 9).

After 6 h of HCK adhesion, cells were observed on all samples with fungal infection, and *C. albicans* appeared as blue dots, smaller than the cell nuclei. The morphology of the HCKs was similar to that seen under monoculture conditions in Fig. 6A (Fig. 8A). Surface covered by HCKs decreased below 50% in fungus-infected SB samples, while adhesion in all ion-implanted samples increased. This indicates that in the samples implanted with Ag and Cu, the presence of early fungal infection did not inhibit HCK adhesion (Fig. 8B). Quantification of the cell area in samples contaminated with *C. albicans* showed values comparable to those in HCK monoculture (Fig. 6C), suggesting that early fungal presence does not significantly affect cell spreading. Additionally, the finding that Cu enhances HCK spreading in short-term exposure was corroborated (Fig. 8C). FESEM micrographs confirmed that *C. albicans* was more prevalent in SB samples, whereas in ion-implanted samples, less surface area was covered by fungi, and a higher presence of HCKs was noted. Cell size was larger in Cu-implanted conditions, consistent with previous findings. FESEM images also revealed that HCKs tended to adhere to areas previously occupied by *C. albicans* rather than directly on the Ti6Al4V surface (Fig. 8F).

In the co-culture with *P. aeruginosa*, HCKs were present on all samples but were less spread compared to those in co-culture with fungi (Fig. 8A). Surface coverage decreased after 2-h *P. aeruginosa* infection, highlighting the detrimental effect of *P. aeruginosa* on HCK adhesion. This decrease was less pronounced in the Ag monoimplanted condition, underscoring the superior short-term antibacterial activity of Ag ions observed in Fig. 6B (Fig. 8D).

A decrease in area was observed across all conditions, with HCKs appearing more rounded, indicating impaired spreading (Fig. 8E). In FESEM micrographs, the presence of *P. aeruginosa* was notably high in the SB condition, almost unnoticeable in the Ag condition, and less prevalent in the Cu and AgCu conditions. The bacteria appeared damaged with undefined walls in the ion-implanted conditions, indicating an effective antibacterial response. Despite this, no significant changes were observed in the size of the HCKs, suggesting that the antibacterial activity did not adversely affect cell biocompatibility. Interestingly, FESEM images revealed that the HCKs were slightly larger in the Ag-implanted samples, implying that Ag ions do not negatively affect cell spreading. These observations confirm the previous results, demonstrating the antifungal effect of Cu, the antibacterial effect of Ag, and the combinatory antimicrobial effect of both when used together (Fig. 8F).

##### Twenty-four-hour co-culture

Immunohistochemistry images after 24-h infection with *C. albicans* showed cells in all conditions, with less spread morphology. *C. albicans* appeared as clusters of small blue dots, with higher prevalence in SB and Ag conditions (Fig. 9A). Surface covered by HCKs decreased in fungus-infected SB samples after 24 h, indicating the detrimental effect of prolonged exposure to *C. albicans* on HCK adhesion (Fig. 9B). The cell area was reduced compared to that of the 6-h co-culture, indicating that extended exposure to *C. albicans* inhibits cell spreading (Fig. 9C). FESEM images revealed that the surface of the SB samples was almost completely covered by *C. albicans*. In the SB and Ag conditions, areas densely populated with filamentous *C. albicans* were observed.

In contrast, the AgCu and Cu conditions exhibited lower presence of *C. albicans*, with some areas entirely free of the fungus and a higher prevalence of the yeast phenotype. Dense hyphal growth made it challenging to identify cells in the FESEM images, with cells visible only in select images, such as those from the Cu condition. These observations underscore the rapid progression of *C. albicans* pathogenicity (Fig. 9F).

In the 24-h co-culture with *P. aeruginosa*, cells were present on all samples, exhibiting the same phenotype observed in the 6-h co-culture (Fig. 9A). For samples infected with *P. aeruginosa*, surface coverage remained similar to that observed in the 6-h co-culture (Fig. 9D). In the presence of *P. aeruginosa*, the HCK area continued to decrease due to prolonged exposure to bacteria (Fig. 9E). For surfaces infected with *P. aeruginosa* for 24 h, FESEM images revealed bacteria on all surfaces. The bacteria appeared damaged with undefined walls in the ion-implanted conditions. Moreover, the ion-implanted samples exhibited fewer bacteria and less cell damage compared to the SB condition, where cells showed extensive damage due to bacterial effects (Fig. 9F).

## Discussion

In this study, metallic ions Ag and Cu or a combination of both were implanted into Ti6Al4V samples using MEVVA ion implantation. The implantation was successful, with the presence of ions confirmed on both the surface and within the bulk of the material. The incorporation of metallic atoms was higher in monoimplanted samples. Biological characterization showed no short- or long-term harmful effects of the implanted surfaces on HCKs. The Ag-implanted samples demonstrated a bactericidal effect, the Cu-implanted samples exhibited an antifungal effect, and the coimplanted samples showed both effects. The co-culture experiments highlighted the superior performance of ion-implanted samples in a preimplantation infection scenario.

FESEM and EDX analyses confirmed that the sandblasting process created an irregular topography on all samples. While Ag and Cu ion implantation did not significantly alter the microroughness generated by sandblasting, it did induce a notable increase in nanoroughness due to sputtering during the implantation process. EDX analysis further confirmed the effective introduction of Ag and Cu atoms.

XPS surface analyses demonstrated through survey spectrum acquisition and surface concentration quantification that Ag and Cu ions were effectively implanted. The chemical states of the implanted atoms varied depending on whether the ions were monoimplanted or coimplanted (Fig. 2B). In coimplanted samples, Ag appeared predominantly in the metallic form, whereas in monoimplanted samples, it was present as a mixture of metal and oxide forms, as reported in previous works [31]. Similarly, Cu was found in both oxide and metallic states in monoimplanted samples but was primarily metallic in coimplanted samples [33]. These findings suggests that the ion implantation process creates a distinct surface chemistry depending on the type and combination of used ions.

The XPS depth profiling showed that both Ag and Cu ions penetrated into the bulk material, with higher surface and bulk concentrations observed in the monoimplanted samples. The profiles followed a Gaussian distribution, attributable to the presence of variously charged copper and silver ions in the plasma generated by cathodic arc discharge.

Ion release studies showed that in the initial hours, a higher surface concentration favored greater release of both Ag and Cu. As the days progressed, ions closer to the surface were released from the bulk of the material. The different release rates were linked to the changes in microstructure introduced after the ion implantation process. These results are positive, as all samples exhibited sustained ion release over several days, which has been associated with preventing acute toxicity while retaining antimicrobial effectiveness [37].

Cell adhesion assays revealed that HCKs adhered well to all ion-implanted surfaces. During the first 6 h, Cu-implanted samples promoted enhanced cell spreading, indicating that Cu ions might initially facilitate better spreading. However, by 24 h, this effect was reversed, aligning with known effects of Cu on various cellular processes. While specific effects of Cu concentrations on HCKs are not well-defined, concentrations below 232.58 ppb seem to support early cell spreading. The observed decrease in HCK area, despite no significant reduction in cell number, may reflect cellular adaptation to Cu rather than cuproptosis [38].

Proliferation assays demonstrated that ion-implanted surfaces supported HCK proliferation over time. Notably, the highest proliferation rate was observed in the Ag monoimplanted condition, which also exhibited the lowest ion release (Fig. 4B).

This suggests that Cu ion release is less favorable for HCK proliferation compared to Ag. However, both Cu and Ag, whether used separately or together, enhance HCK proliferation on Ti surfaces. These results align with previous studies showing improved HCK proliferation in the presence of Ag ions, although the exact mechanisms remain unclear [39]. The Cu monoimplanted condition showed lower proliferation compared to the other ion-implanted conditions, but it still outperformed the SB control.

Dysregulation of intracellular copper levels can induce oxidative stress and potentially lead to cuproptosis [40]. Despite an initial decrease in cell area triggered by Cu, these morphological changes did not significantly impair cell growth. This suggests that the Cu concentrations present in the Cu monoimplanted and AgCu conditions do not adversely affect HCK proliferation.

The cytocompatibility studies with HCKs confirmed that ion-implanted surfaces supported both cell adhesion and long-term proliferation, including under Cu-implanted conditions. This highlights the importance of maintaining balanced Cu levels to prevent adverse effects, such as cuproptosis, and to ensure cellular health. With cytocompatibility established, the next step was to investigate the antimicrobial performance of the ion-implanted surfaces.

The 2-h adhesion assays demonstrated that ion-implanted surfaces effectively combated *P. aeruginosa* and *C. albicans*, 2 common pathogens linked to BKPro-related infections. Ag-implanted surfaces showed clear early antibacterial activity through reduced *P. aeruginosa* adhesion and viability. FESEM images confirmed bacterial damage on Ag-implanted samples. These findings suggested membrane disruption and ROS generation as Ag’s early antibacterial acting mechanism [41]. In the early hours, Cu-implanted surfaces significantly reduced fungal adhesion and viability, as evidenced by FESEM images that showed fewer *C. albicans* cells and a reduced number of pseudohyphae. These findings indicated impaired vital enzymatic functions and ROS generation as early antifungal mechanism of Cu [42].

Kinetic growth assays demonstrated the sustained antifungal activity of Cu, effectively reducing the fungal population in both the Cu monoimplanted and AgCu conditions (Fig. 7E). Similarly, Ag monoimplanted, AgCu, and Cu monoimplanted surfaces exhibited sustained antibacterial activity (Fig. 7F). In this study, PB was used to assess microbial growth by measuring metabolic activity, a reliable indicator of cell viability [43]. A decrease in metabolic activity indicates a reduction in the microbial population, suggesting a potential prevention of biofilm formation [35]. Ag and Cu ions showed notable, sustained antibacterial activity against *P. aeruginosa*, in line with previous research that emphasizes the ability of Ag and Cu ions to disrupt bacterial communication and adhesion processes, crucial in biofilm development, and their known capacity to inhibit biofilm development [44].

The distinct antimicrobial efficacy of Ag, Cu, and AgCu ion-implanted surfaces results from their complementary mechanisms. Ag ions disrupt bacterial membranes, bind to protein thiol groups, and interfere with DNA replication and enzymatic functions, making them particularly effective against *P. aeruginosa*. Cu ions exhibit strong antifungal activity by generating ROS and disrupting cell homeostasis, with moderate antibacterial effects through similar oxidative stress pathways.

The combination of Ag and Cu ions provides a broader and more sustained antimicrobial effect. This synergy arises from the direct bactericidal actions of Ag and the oxidative stress contributions of Cu, potentially preventing microbial resistance by targeting pathogens with multiple mechanisms [11].

The delayed regrowth and prolonged antimicrobial activity observed in the AgCu condition suggest that the combination of Ag and Cu offers continuous protection against both bacterial and fungal colonization on titanium surfaces.

The preimplantation infection co-culture model is crucial for accurately assessing antimicrobial surfaces, as it replicates the clinical scenario of infection prior to implantation. This method provides a realistic evaluation of the effectiveness of the surfaces in preventing microbial adhesion and biofilm formation and maintaining biocompatibility [26]. In this study, the ion-implanted surfaces were tested using this co-culture strategy. The obtained results demonstrated that ion-implanted surfaces effectively preserved their antimicrobial properties while supporting HCK adhesion, even in the presence of infection. Ag-implanted surfaces significantly enhanced bacterial inhibition. Cu-implanted surfaces maintained HCK surface coverage levels comparable to those in non-infected conditions even in fungus-infected environments, while AgCu-implanted surfaces exhibited the combined effects of both ions.

The early presence of *C. albicans* did not significantly impair cell adhesion or spreading on Ag- and Cu-implanted surfaces, in contrast to control surfaces where fungal presence caused substantial decreases in cell coverage at initial hours. After 24 h, the Cu and AgCu conditions demonstrated reduced fungal presence and enhanced cell coverage compared to the control and Ag conditions. Furthermore, the absence of visible fungal damage in FESEM images, combined with the decreased number of fungi and reduced hyphal structures on Cu-implanted surfaces, aligns with previous studies. These studies revealed that copper repletion prompts *C. albicans* to down-regulate genes associated with copper uptake and energetic pathways critical for survival. Such adaptations lead to a reduction in *C. albicans* virulence, as the yeast-to-hypha transition, essential for its pathogenicity, is inhibited [45]. This underscores the crucial role of copper homeostasis in regulating fungal virulence.

In the co-culture with *P. aeruginosa*, ion-implanted surfaces exhibited reduced bacterial presence and, therefore, cell damage. Although cells in the presence of *P. aeruginosa* remained poorly spread, this was less severe on ion-implanted surfaces compared to the control, indicating improved conditions despite ongoing bacterial impact. These results are promising, especially given the previously reported severity and threat of *Pseudomonas* infections in HCKs [46].

The results obtained from co-culture experiments are significant, as they reflect realistic implantation conditions. This underscores the value of preimplantation infection co-culture models in the evaluation of biomaterials [25]. The present study represents the first co-culture investigation of HCKs with *P. aeruginosa* and *C. albicans*, offering new insights into cell–pathogen interactions and the efficacy of ion-implanted surfaces, underscoring the novelty and significance of this research.

The use of metallic ions in biomedical implants represents a promising strategy for combating infections and enhancing implant longevity and functionality [11]. Ag and Cu ions are effective antimicrobial agents, reducing the risk of postsurgical infections by preventing bacterial and fungal adhesion and proliferation on implant surfaces [47].

This is especially beneficial for high-risk patients or environments prone to microbial contamination. Additionally, these ions appear to be nontoxic to corneal tissue, as they promote cellular adhesion and proliferation, thereby enhancing implant integration. However, potential risks must be managed, as excessive release of Ag and Cu ions can cause cytotoxic effects, harming surrounding tissues and disrupting normal cell function [48].

Achieving an optimal balance between antimicrobial activity and minimal cytotoxicity requires precise control of ion concentration and release rates. Therefore, while Ag and Cu offer significant advantages in infection prevention, their use must be carefully optimized to ensure biocompatibility and minimize potential adverse effects.

In recent years, the European Union has tightened regulations concerning the use of Ag and Cu in consumer products, cosmetics, and medical devices to safeguard public health and the environment [49,50]. Our study supports this initiative by demonstrating that the ion implantation of Ag and Cu onto Ti surfaces, using the MEVVA ion implantation technique, results in significantly low ion release while retaining strong antimicrobial efficacy. Despite the potential toxicity risks of metallic ions, our findings reveal minimal ion release with excellent cytocompatibility with HCKs, indicating a reduced risk. Furthermore, we showed that these low ion release concentrations of Ag and Cu are still effective against microbial strains commonly linked to ophthalmic infections while ensuring HCK compatibility.

The findings of this study suggest that Ag and Cu ion implantation through the MEVVA ion implantation technique can effectively enhance the antimicrobial properties of the Ti BKPro backplate while maintaining biocompatibility. The varying ion release rates and specific activities against bacteria and fungi offer tailored strategies for addressing different types of infections.

## Conclusion

Ion implantation was used to implant Ag, AgCu, and Cu ions onto rough Ti surfaces. On surfaces implanted with a single type of ion, these ions were found in metallic and oxide forms, whereas on surfaces implanted with both ions, Ag and Cu were found only in metallic form. The absence of harmful effects on the adhesion and proliferation of HCKs was confirmed. Ag-implanted surfaces exhibited a significant bactericidal effect against *P. aeruginosa*, while Cu-implanted surfaces showed antifungal activity against *C. albicans*. AgCu samples demonstrated a combination of both effects, highlighting the synergistic action of the 2 ions in providing enhanced antimicrobial defense. Despite the low release of ions, this study demonstrates that the obtained surfaces exhibit clear antimicrobial properties and good compatibility with corneal tissue. This study offers valuable insights into the antimicrobial properties and biocompatibility of Ag, AgCu, and Cu ion-implanted Ti surfaces. However, future research should validate these findings through in vivo experiments and evaluate the long-term efficacy and safety of ion-implanted surfaces. Additionally, future studies should explore patient-specific responses to metallic ion implantation. These findings underscore the potential for these surfaces to be used as replacements for the classic Ti backplate of BKPro, improving biointegration and reducing infection rates.

## Data Availability

Data will be made available on request.
